# MicroRNA Expression in Cystic Fibrosis Airway Epithelium 

**DOI:** 10.3390/biom3010157

**Published:** 2013-02-11

**Authors:** Catherine M. Greene

**Affiliations:** Respiratory Research Division, Department of Medicine, Royal College of Surgeons in Ireland, Education and Research Centre, Beaumont Hospital, Dublin 9, Ireland; E-Mail: cmgreene@rcsi.ie; Tel.: +353-1-809-3800; Fax: +353-1-809-3808

**Keywords:** cystic fibrosis, airway epithelium, microRNA

## Abstract

MicroRNAs (miRs) have emerged as major regulators of the protein content of a cell. In the most part, miRs negatively regulate target mRNA expression, with sets of miRs predicted to regulate certain signaling pathways. The miR expression profile of endobronchial brushings is altered in people with cystic fibrosis (CF) compared to those without CF. How this impacts on CF has important implications for our growing understanding of the pathophysiology of CF lung disease and the development of new therapeutics to treat its pulmonary manifestations. Herein we discuss the potential consequences of altered miR expression in CF airway epithelium particularly with respect to cystic fibrosis transmembrane conductance regulator (CFTR) expression, innate immunity and toll-like receptor signalling and explore how best to exploit these changes for therapeutic benefit.

## 1. Introduction

### 1.1. Cystic Fibrosis

Cystic fibrosis is a multisystem disorder affecting many organs including the liver, intestines, reproductive tract, bone, heart, spleen, gall bladder and pancreas. The respiratory tract is also affected in cystic fibrosis and it is the pulmonary manifestations of the disease that are responsible for the associated high morbidity and mortality in people with CF [1]. This is an autosomal recessive genetic disease caused by mutations in the gene encoding the cystic fibrosis transmembrane conductance regulator (CFTR) protein, a cyclic AMP activated chloride ion channel expressed in the apical membranes of epithelial cells in the lung and other organs. In excess of 1800 *CFTR* mutations have been identified, many of which are associated with disease. These mutations can be grouped into six classes (I-VI) depending on whether the mutations affect the quantity or function of CFTR, or a combination of these [2]. ΔF508 CFTR is the most common mutation worldwide and belongs to class II. Due to aberrant CFTR expression and/or function the CF lung develops unusual physiological characteristics. Firstly, the volume of the airway surface liquid covering the airway epithelium is decreased. This is associated with hypersecretion of dehydrated viscous mucus and an impaired mucociliary escalator. Together with a high salt, low pH and proteolytically active environment, conditions for the colonization and growth of microorganisms are established. The major bacterial pathogens found in the CF lung are *Staphylococcus aureus*, *Pseudomonas aeruginosa*, *Burkholderia cepacia* complex, and *Stenotrophomonas maltophilia.* Some of these can adopt a biofilm mode of growth and are particularly difficult to eradicate. Anaerobes such as *Prevotella* and *Viellonella* species and *Streptococcus milleri* are increasingly recognised as emerging CF pathogens along with atypical mycobacteria. In addition to bacteria, fungi are also able to colonize the CF lung; *Candida albicans* and *Aspergillus fumigatus* are amongst the most frequently isolated. Together with *CFTR* genotype, gender, co-morbidities and other organ involvement these features all contribute to CF disease progression and severity. Treatment regimens and exacerbation history also have an impact.

In the U.S. the median age of survival for people with CF has increased from 31 to 37 years over a recent 10-year period. In the U.K., those born with CF now are predicted to have a median life span of approximately 50 years. These improvements are due to a combination of our enhanced understanding of the pathophysiology of CF and the discovery and introduction of CF-specific therapies. The current broad ranging treatments include, amongst others, physical therapies and nutritional supplementation strategies, mucolytics, anti-inflammatories and antibiotics [3]. Notwithstanding the significant therapeutic advances that have been made it is important to investigate and develop alternatives to these existing strategies that may have unique and improved therapeutic effects.

The bronchial epithelium plays a particularly important role in CF. In addition to providing a physical barrier against invading microbes, the CF epithelium responds to the changing pulmonary environment with the expression of soluble and secreted factors such as cytokines and antimicrobials. A striking feature of CF lung disease is the abnormally high level of infiltrating neutrophils. These cells secrete proteases and oxidants that, together with other CF-specific lung agonists, can exacerbate inflammation by inducing expression of interleukin-8 (IL-8) [4,5,6,7,8]. IL-8 is abundantly expressed by CF bronchial epithelial cells and is a potent neutrophil chemokine. Understanding the behavior of the airway epithelium in the CF lung and determining how alterations in its biology due to intrinsic defects or infective insults can impact on CF may lead to the identification of new therapeutics.

### 1.2. MicroRNAs

MicroRNAs are short endogenous non-coding single-stranded RNA oligonucleotides. miRs modulate target gene expression via translational repression or degradation of their target mRNAs. They mediate these effects by binding, with a complex of proteins called the RNA-induced silencing complex (RISC or miRISC), to complementary or partially complementary six to eight nucleotide sequences termed miR recognition elements (MREs) encoded in the primary sequence of target mRNAs. Once localized to the target mRNA translation can be inhibited by blocking ribosomes or mRNA degradation can occur via the ribonuclease activity of argonaute proteins within RISC. miRs are implicated in several biological processes and disease states and their expression levels vary greatly among species and tissues. Dysregulation of miR expression is known to contribute to the pathology of a number of diseases. Thus these regulatory RNAs that assist in controlling the protein content of a cell may represent a new and important class of drug targets. Therapeutic modulation of miRs is possible by the use of double-stranded miR mimetic overexpression (premiRs) or antisense inhibition by antagomirs [9].

## 2. MicroRNA Expression Profiling Studies in Cystic Fibrosis Bronchial Epithelium

**Figure 1:**
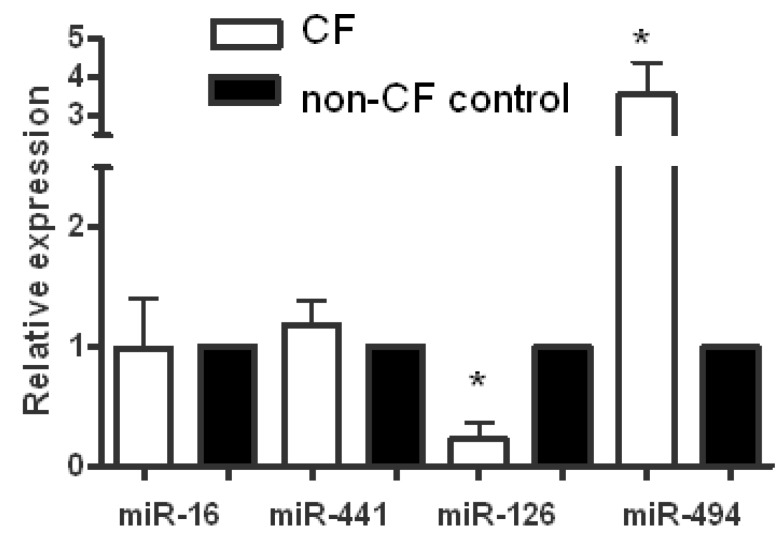
MicroRNAs (miR) expression *in vivo*. Expression levels of miR-16, miR-441-5p, miR-126 and miR-494 in cystic fibrosis (CF) (white bars) and non-CF (black bars) bronchial brushings measured by TaqMan Low Density array (CF and control; n = 5 each). Data are represented as fold change compared to normalized controls (*p < 0.05).

Oglesby *et al*. performed the first miR expression profile studies in CF [10]. They set out to investigate if unique miR expression profiles exist in CF airway epithelium, to identify and validate functional targets of important miRs identified in the screening studies and to assess their role in airway epithelial biology and inflammation in the CF lung. Their miR profiling studies were performed on endobronchial brushings from five individuals with CF and five non-CF controls using the Taqman MicroRNA Low density Arrays v2.0 (released June 2009) from Applied Biosystems. The miR content in these arrays is derived from the miRBase microRNA registry, providing comprehensive coverage of miRs from release 10.0 using the most up-to-date TaqMan MicroRNA Assays. Following reverse transcription and cDNA amplification, the samples were run on two array cards on the Applied Biosystems 7900HT fast real time PCR system and relative quantification of miR expression was determined using the comparative cycle threshold method (2^(−ΔΔCt)^). At the time this system allowed the direct quantification of 667 different human miRs in each sample. Whilst the expression of a number of miRs such as miR-16 and miR-441-5p were not significantly different in CF *versus* non-CF samples they did observe significant decreases and increases in expression of specific miRs (Figure 1). For example, miR-126 was decreased in CF samples compared to controls whereas miR-494 was increased in the CF vs. non-CF samples. Overall they identified 92 differentially expressed miRs between the two cohorts; in the CF samples 56 miRs were decreased less than 0.7 fold and 36 were increased greater than 1.5-fold. Many of these miRs were predicted to target components of pathways important in CF lung disease.

The next study was carried out by Bhattacharyya *et al*. [11]. This group used the CF and non-CF bronchial epithelial cell lines IB3-1 and IB3-1/S9, respectively to identify 22 differentially expressed miRs; 18 were elevated and 4 were reduced in the CF vs. non-CF cells. They used a hybridization approach that involved probing Ambion miR expression arrays with [γ -^32^P]dATP-labeled total RNA from either cell type, capturing the signals using a phosphorimager and quantifying the data using ImageQuant software. They validated these observations using individual TaqMan qPCR miR assays. There were no major similarities between the expression profiles from the *ex vivo* and *in vitro* studies however this could be due to the use of different source materials (patient samples *versus* cell lines), profiling methodologies (*in situ* qPCR *versus* hybridization) and miR representation on the different platforms (667 *versus* 365). The major discrepancies between the two studies are the results regarding miR-23b and miR-126: these miRs were upregulated in CF cell lines but down-regulated in CF bronchial brushings. Our group have since further validated the expression and function of these miRs in additional patient samples and CF and non-CF epithelial cells lines and confirm that both miR-23b and miR-126 are decreased *in vivo* in the CF lung ([10], and K Gaughan, personal communication). Overall the changes in miR expression in CF epithelium are likely due to altered ion transport, endoplasmic reticulum stress, infection and inflammation.

Other than these two reports, there appear to have been no other miR profiling studies performed on human CF bronchial epithelial cells to date. Bazett *et al.* investigated the miR expression profile in intestinal tissue from CFTR deficient mice compared to their wild type littermates [12]. Their work identified 24 miRs that influence CF intestinal disease and suggests that miR-based medicines may also have potential in the treatment of the non-pulmonary manifestations of CF.

## 3. microRNA Regulation of CFTR Expression

The *CFTR* gene locus spans 189kb and encodes a 6.2kb mRNA transcript with a 3’UTR of 1556 bases. Expression of *CFTR* is regulated in a temporal and tissue-specific manner via *cis*-acting genomic elements however post-transcriptional regulation is also important. In addition to alternative splicing, regulation by miRs is now known to control expression of CFTR. A number of groups have investigated this concept [13,14,15,16]. Whilst indirect regulation of CFTR expression can be achieved by miRs that target components of a cell’s transcriptional regulatory machinery [15], direct targeting of CFTR by miRs is also possible. Using TargetScan 5.0 Gillen *et al.* identified 106 miRs predicted to target the CFTR 3’UTR. Focusing on 13 of these candidate miRs they studied the effects of premiR mimics on (i) CFTR mRNA expression, (ii) CFTR protein expression, and (iii) a CFTR 3’UTR luciferase reporter gene. Their observations indicated that miRs can indeed regulate expression of *CFTR* with miR-145 and miR-494 being the most effective [13]. A second study also identified miR-494 from a list of 496 putative CFTR targeting miRs as being important [15]. However, unlike the previous work which saw no effect of miR-101 on expression of luciferase from a full length CFTR 3’UTR reporter construct, this group saw an inhibitory effect of premiR-101 using a truncated 741 bp CFTR 3’UTR reporter construct. Independently another group provided more evidence for a role for miR-101 (and miR-144, each of which binds to the same site in the CFTR 3’UTR) in regulation of CFTR mRNA and protein expression [16], however they suggested that these miRs may have a role in cigarette-smoke-induced inhibition of CFTR in the context of chronic obstructive pulmonary disease. 

Our group has investigated the role of miRs in CFTR regulation *in vivo* in bronchial brushings from individuals homozygous or heterozygous for ΔF508 CFTR [17]. We saw no role for miR-101 however miR-145, miR-223 and miR-494 all of which are predicted to target the CFTR 3’UTR were upregulated in CF *versus* non-CF bronchial brushings and cell lines. Following manipulation of these three miRs using premiRs or antimirs, we observed reciprocal down or up regulation of CFTR gene and protein expression; CFTR function was similarly affected. Using a reporter system containing a wild type or mutated full length CFTR 3’UTR we demonstrated direct miR/target relationships. Increased *in vivo* expression of miR-145, miR-223 and miR-494 correlated with carriage of the ΔF508 CFTR mutation and colonization with *Pseudomonas aeruginosa*, but not other organisms. The data indicate that defective chloride ion conductance, inflammatory and infective insults regulate miR-145, miR-223 and miR-494 expression in ΔF508 CFTR bronchial epithelial cells and contribute to altered ΔF508 CFTR expression. Overall the strongest evidence points to miR-145 and miR-494 as major regulators of CFTR, with miR-223 also being important in the context of ΔF508 CFTR.

## 4. Innate Immunity in Cystic Fibrosis

Pulmonary innate immunity comprises many aspects ranging from the cells that form the epithelial barrier and carry out phagocytosis and bacterial killing, to soluble cytokines, antimicrobials and antiproteases, and cell surface receptors that can recognize and discriminate pathogens. In the CF lung many innate immune mechanisms are abnormal and this contributes to the excessive pulmonary inflammation and dysregulated airway physiology associated with the disease. This is a vast research area that has been reviewed recently elsewhere [18]. Here we present two examples of how altered miR expression in CF can impact on pulmonary innate immunity.

### 4.1. Role of miR-126 in Innate Immunity in CF Bronchial Epithelium

Expression of miR-126 is high in vascularised tissues such as the lung, heart and kidney and it is known to be expressed in bronchial epithelium. miR-126 is 21 nucleotides in length, is encoded on chromosome 9q24 and is contained within intron 5 of the host gene epidermal growth factor like-7 (EGFL-7). Based on the observations of our miR expression profiling studies we investigated whether miR-126 was also differentially expressed in CF *versus* non-CF airway epithelial cell lines by performing qRT-PCR on CF tracheal and bronchial epithelial cell lines and their non-CF counterparts. This revealed that miR-126 is significantly downregulated in CF compared to non-CF bronchial epithelial cells. We also determined miR-126 levels in a variety of cell lines by qRT-PCR and observed higher expression of miR-126 in lung airway epithelial *versus* non-lung cells including monocytic, fibroblast, hepatoma and astrocytoma cells.

In order to identify potential targets of miR-126 both for experimental validation and functional studies in airway inflammation *in silico* analysis of a range miR target prediction databases was performed. These searches pointed to various predicted targets of miR-126 including Tsc1 and TOM1. Focussing on TOM1 RNA Hybrid calculated the predicted binding between the seed region of miR-126 and the miR-126 MRE within the TOM1 3’UTR as having a minimum free energy (mfe) of −21.5 kcal/mol and notably TargetScan 4.2 showed that the predicted pairing region in the TOM1 3’UTR was conserved across species. When we measured TOM1 expression in CFBE41o^– ^*versus* 16HBE14o^– ^cell lines by qRT-PCR we saw a significant increase in TOM1 expression in the CF cells line and a clear reciprocal relationship between TOM1 and miR-126 levels in both cell lines. We also assessed TOM1 mRNA levels in the original bronchial biopsies that were used for the profiling studies and were able to confirm the *in vitro* studies. Non-CF cells expressed approximately 50% lower levels of TOM1.

TOM1 is involved in intracellular trafficking. It has been proposed to be a negative regulator of IL-1β and TNF-α signalling whereby its overexpression was shown to suppress activation of the transcription factors NFκB and AP1. TOM1 forms a complex with Tollip, which is involved in IL-1R1 and TLR signaling and this complex has been shown to traffic the IL-1R to the endosome for degradation. Our group has a particular interest in the TLR/IL-1R superfamily, particularly in the context of CF epithelial cells [4,5,6,7,8,19,20,21]. With this as background we confirmed that TOM1 is a molecular target of miR-126 using a luciferase reporter vector containing the full-length TOM1 3'-UTR. HEK293 cells, which exhibit low levels of miR-126 expression, were used for transient transfections with pMIR-TOM1-3’UTR. Co-transfection with premiR-126 resulted in a significant decrease in luciferase gene expression from the reporter vector containing the TOM1 3'-UTR when compared to a scrambled control demonstrating direct targeting by miR-126. Over-expression of miR-126 decreased TOM1 protein in CFBE41o^–^ cells.

In order to determine functional effects of TOM1 in the context of the CF lung we transfected CFBE41o^–^ cells with a TOM1 over-expression plasmid, and assessed its effects on NFκB in these cells in response to inflammatory stimuli common in the CF lung utilizing an NFκB reporter system. NFκB reporter gene expression in CFBE41o^–^ cells was measured in response to stimulation with LPS or IL-1β. The agonists significantly increased NFκB reporter gene expression compared with controls, whilst over-expression of TOM1 inhibited this effect. IL-8 is an NFκB regulated gene and a key cytokine present in the CF lung. We assessed the effect of TOM1 knockdown on IL-8 protein production in CFBE41o^–^ cells. IL-8 levels were measured in response to stimulation with the TLR2 and TLR4 agonists lipopeptide and LPS, or IL-1β. Each of these agonists significantly increased IL-8 protein production compared with untreated cells. TOM1 knockdown potentiated the stimulatory effects of all with IL-8 secretion significantly increased. Together these studies provided a functional role for TOM1 in TLR2, TLR4 and IL-1β the signaling pathways and link this observation with regulation of TOM1 by miR-126. This was the first report of miR involvement in CF. This and other ongoing studies in our group are examining the roles of miRs in innate immune responses in the CF lung and are aimed at developing new miR-based medicines to treat the pulmonary manifestations of CF. 

### 4.2. miR-Mediated Regulation of Interleukin-8 in CF Bronchial Epithelium

IL-8 is a neutrophil chemokine. Bronchial epithelial cells and macrophages in the lung can express IL-8 but given that CF is a bronchial disease and due to the vast surface area of the bronchial epithelium, these cells represent the major source of IL-8 in the CF lung. The large numbers of neutrophils that accumulate within the CF lung in response to IL-8 secrete proteases and oxidants that lead to derangement of the lung, overwhelm the normal anti-protease and anti-microbial defenses of the respiratory epithelial surface and promote proinflammatory gene expression [22]. Thus neutrophils and their products play a major role in pulmonary inflammation in CF. Although we performed the original miR profiling studies in CF *versus* non-CF bronchial epithelium, the role of miRs modulating IL-8 was not addressed there [10]. In fact miR studies related to IL-8 expression are relatively few. Three studies have reported how miRs targeting RelA (miR-530/373) [23], IKKβ (miR-199a) [24] or SHIP1 (miR-155) [11] can indirectly impact on IL-8 gene expression. Other studies in this area include two reports implicating miR-146a/b in IL-8 regulation [25,26] and another on miR-132 which also only indirectly regulates IL-8 [27]. Hu *et al*. have demonstrated that IL-8 is susceptible to modulation by miR-520b in breast cancer cells using premiRs and antimirs [28], whilst Yu *et al.*, in the context of breast cancer metastasis, have shown that the miR-17/20 cluster regulates the IL-8 3’UTR [29]. Although a number of therapeutic approaches in CF indirectly lead to decreased cytokine expression [20,30,31,32,33,34] there have been no studies to date that directly target IL-8 expression in the CF lung and none using miR modulators. 

In addition to IL-8, leukotriene B4 (LTB4) and other less abundant chemotactic peptides are also present in the CF lung. In the past strategies targetting LTB4 in CF had too potent an inhibitory effect and were unsuccessful [35]. The reasons for this are that the LTB4 antagonists used not only impaired neutrophil chemotaxis in response to LTB4 but also caused neutrophil apoptosis thereby killing whatever neutrophils managed to migrate to the lung in response to other chemokines [36]. The goal in controlling the excessive neutrophil infiltration into the CF lung is to restore chemotaxis to normal rather than sub-normal levels. In this regard our goal is to inhibit abnormal expression of IL-8 whilst leaving intact the normal processes which are necessary and sufficient for physiological neutrophil infiltration into the lung. 

Transcriptional regulation of IL-8 in CF bronchial epithelium is complex. Strategies designed to interfere with IL-8 gene transcription are likely to have therapeutic potential for CF however interfering with its post-transcriptional regulation is also possible. We are investigating the therapeutic potential of miR-based medicines for CF. A search for miRs that target IL-8 was performed *in silico* and a shortlist of 115 miR candidates was created. By cross-comparing this list with miRs known to be decreased *in vivo* in CF bronchial brushings we have identified 15 lead IL-8-specific miRs. Ongoing studies have narrowed down this list to 4 miRs that together can effectively and selectively inhibit IL-8 expression in a CF bronchial epithelial experimental system. It is envisaged that further work will develop these miRs into effective medicines for treatment of the pulmonary inflammatory manifestations of CF. 

## 5. miR-Based Medicine for Cystic Fibrosis Lung Disease

In order for a new therapy to be effective it must first demonstrate *in vitro* potential. Although few miR-based medicines have reached the clinic, many show promise for further development. Therapeutic modulation of miRs is possible by the use of antisense oligonucleotide approaches for inhibiting miR function and premiR overexpression approaches for increasing miR expression [37,38]. PremiRs are double-stranded miR mimetics that are structurally analogous to siRNAs. Plasmid or viral vectors expressing shRNAs can also be used to overexpress miRs and have the potential for more persistent expression as this approach can facilitate the expression of multiple miRs from one transcript. However delivery of these agents to the lung represents a challenge due to their size and charge (reviewed in [9]). Novel nanotechnologies that overcome these problems and effectively deliver RNA-based therapies to cells, including airway epithelial cells are under development [39]. Such inhalable nanoparticles would facilitate effective miR delivery and target gene knockdown in bronchial epithelial cells with limited cytotoxicity. Thus whilst identifying miR-targetted therapeutics *in vitro* is worthwhile, incorporating strategies to facilitate their delivery to target cells is also needed. A delivery system should enhance bioavailability of the drug by protecting it from degradation or inactivation, target the drug so that it accumulates at the diseased site and optionally, deliver the drug directly to the focus of disease (*i.e*., the lung) [40]. Nanoparticles designed specifically for delivery to airway epithelial cells that can penetrate mucus, facilitate rapid and efficient cell uptake and minimise toxicity would be an effective new therapy. Ideally these could be encapsulated in bioresponsive, inhalable hydrogels to facilitate targetted release of the particles only at sites of inflammation in the lungs following aerosolisation. Once the design, synthesis, physicochemical and biophysical characterization of these nanoparticles is accomplished, their pharmacological activity will need to be specified. This task will require appropriate cellular test systems to study drug release profiles, cellular uptake mechanisms and intracellular trafficking, and the establishment of primary epithelial barriers, which mimic the natural environment of the CF lung for the examination of adhesion, permeation and translocation processes. Next safety and risk must be addressed to exclude potential toxicity or immunogenicity caused by the delivery system and its components [41]. Once validated, the new medicines can then be tested in appropriate *in vivo* models to examine biodistribution and clearance rates. 

## 6. Conclusion and Perspective

Although our knowledge of the role of miRs in cystic fibrosis is still growing, a number of important miRs have now been identified that could be considered for therapeutic development. PremiR medicines based on combinations of miRs that target IL-8 have the potential to decrease IL-8 expression from bronchial epithelial cells and correct the over exuberant neutrophil influx into the CF lung. Conversely by inhibiting expression of various miRs (miR-145, miR-223, miR-494) using antimir approaches, aberrant CFTR expression may be corrected and effective chloride ion conductance could be restored. The major challenges in this regard will be to optimise the efficacy of the chosen miRs/antimirs, reduce their off target effects, and deliver these pharmaceuticals directly to the airways. The next five years are likely to bring major advances in these areas.
